# Fatal Tumor Lysis Syndrome Induced by Pembrolizumab in Advanced Renal Pelvis Cancer

**DOI:** 10.1002/iju5.70097

**Published:** 2025-11-20

**Authors:** Takashi Asakura, Toshiaki Shinojima, Shinnosuke Hiruta, Hirotaka Asakura

**Affiliations:** ^1^ Department of Urology Saitama Medical University Saitama Japan

**Keywords:** pembrolizumab, renal pelvis cancer, tumor lysis syndrome

## Abstract

**Introduction:**

Tumor lysis syndrome (TLS) arises from the rapid breakdown of tumor cells during oncological treatment. Although TLS is rarely observed in solid tumors, few studies have documented instances of TLS associated with pembrolizumab. This report presents a case involving pembrolizumab‐induced TLS.

**Case Presentation:**

A 76‐year‐old male patient with advanced renal pelvis cancer (T3N2M1) was treated with pembrolizumab as second‐line therapy. Four days after the initiation of therapy, the patient developed TLS. Despite intensive therapeutic interventions, he succumbed to the condition on the 10th day.

**Conclusion:**

This case highlights a fatal TLS episode after pembrolizumab. Given its poor prognosis, blood monitoring and prophylaxis are warranted in intermediate‐risk patients, particularly when multiple non‐renal risk factors are present.

**Abbreviations**
ACTH: adrenocorticotropic hormone
CRP: C‐reactive protein
CT: computed tomography
DIC: disseminated intravascular coagulation
ECOG‐PS: Eastern Cooperative Oncology Group Performance Status
Free T4: free thyroxine
ICI: immune checkpoint inhibitor
irAEs: immune‐related adverse events
LDH: lactate dehydrogenase
TLS: tumor lysis syndrome
TSH: thyroid‐stimulating hormone

**Summary**

- With the adoption of potent new therapies as ICI, TLS incidence in solid tumors is expected to rise. Given its poor prognosis, intermediate‐risk cases require close monitoring and prophylaxis.

## 1 Introduction

TLS is a serious condition that arises from the rapid breakdown of malignant tumor cells during treatment. TLS rarely occurs in solid tumors. Moreover, few studies have documented pembrolizumab‐induced TLS. This case report presents the progression of a case involving pembrolizumab‐induced TLS.

## 2 Case Presentation

**FIGURE 1:**
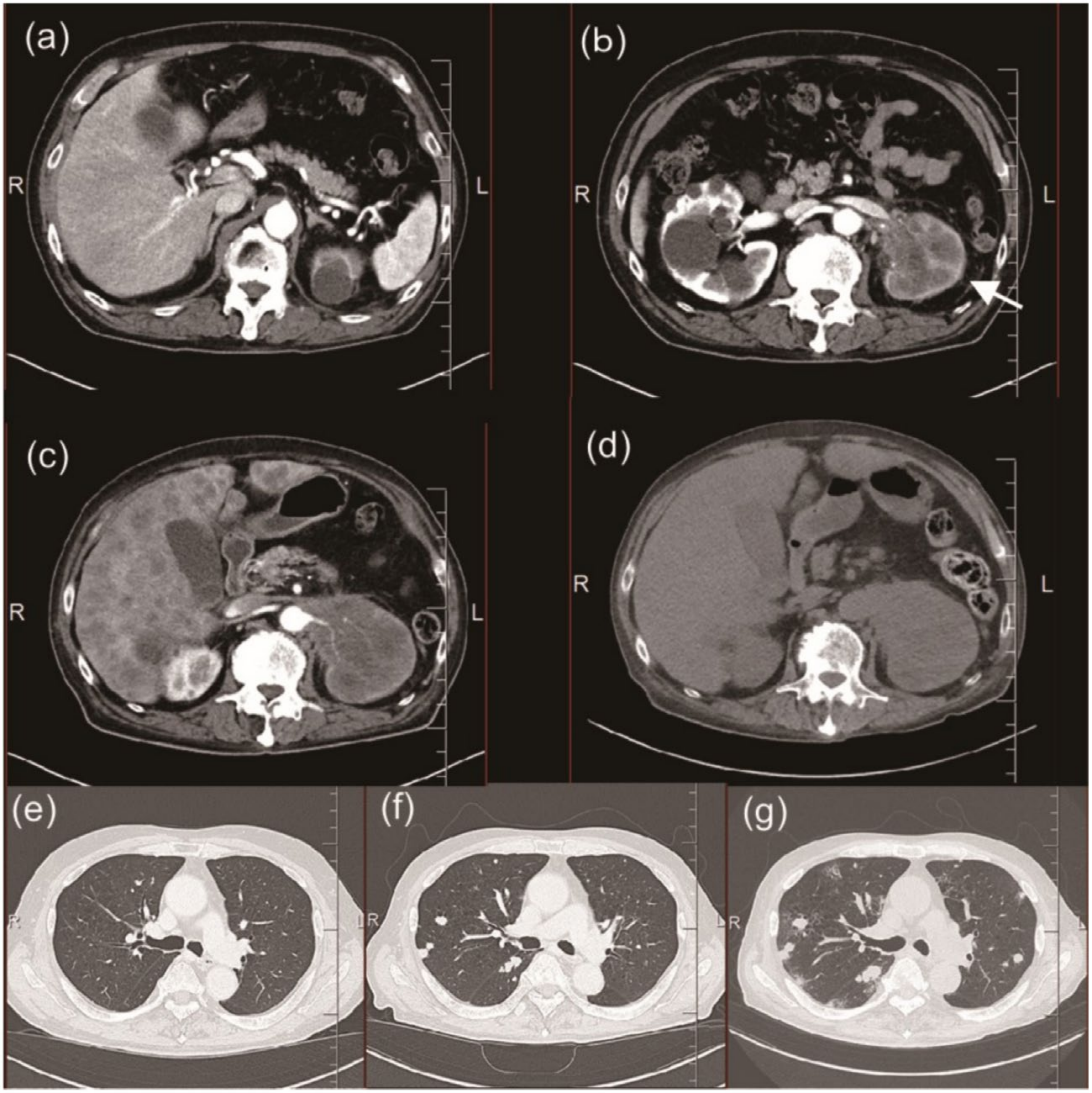
CT images. (a, b) Arterial‐phase CT before the first chemotherapy, with (a) showing the liver level and (b) showing a primary tumor in the left renal pelvis (white arrow). (c) Arterial‐phase CT before pembrolizumab administration demonstrating multiple liver metastases and enlargement of the primary tumor. (d) Plain CT obtained 4 days after pembrolizumab administration showing further hepatomegaly. (e–g) Lung field CT scans taken before initial chemotherapy (e), before pembrolizumab administration (f), and after pembrolizumab administration (g), illustrating progression of lung metastases over time.

A 76‐year‐old male patient with an ECOG‐PS score of 1 presented with gross hematuria. A CT scan revealed a tumor in the left renal pelvis, with enlarged para‐aortic lymph nodes and metastases to the left adrenal gland and liver. A ureteroscopic urine sample was collected from the left renal pelvis, which yielded a cytology result of class V. The patient was diagnosed with advanced renal pelvis cancer (T3N2M1). Initially, the patient was treated with gemcitabine and cisplatin. His prechemotherapy CRP was 1.94 mg/dL and ECOG‐PS was 1. Following two cycles of chemotherapy, a CT scan revealed new lung metastases and increased size of the primary tumor (Figure 1), leading to a diagnosis of progressive disease.

Subsequently, the patient was admitted to the hospital for second‐line therapy with pembrolizumab. His admission blood tests showed renal dysfunction (creatinine, 1.43 mg/dL), hepatic dysfunction (aspartate aminotransferase, 65 U/L; alanine aminotransferase, 36 U/L; and alkaline phosphatase, 513 U/L), elevated CRP, 13.97 mg/dL, and increased LDH, 1039 U/L. Despite a reduction in appetite, his ECOG‐PS score remained at 1.

**FIGURE 2:**
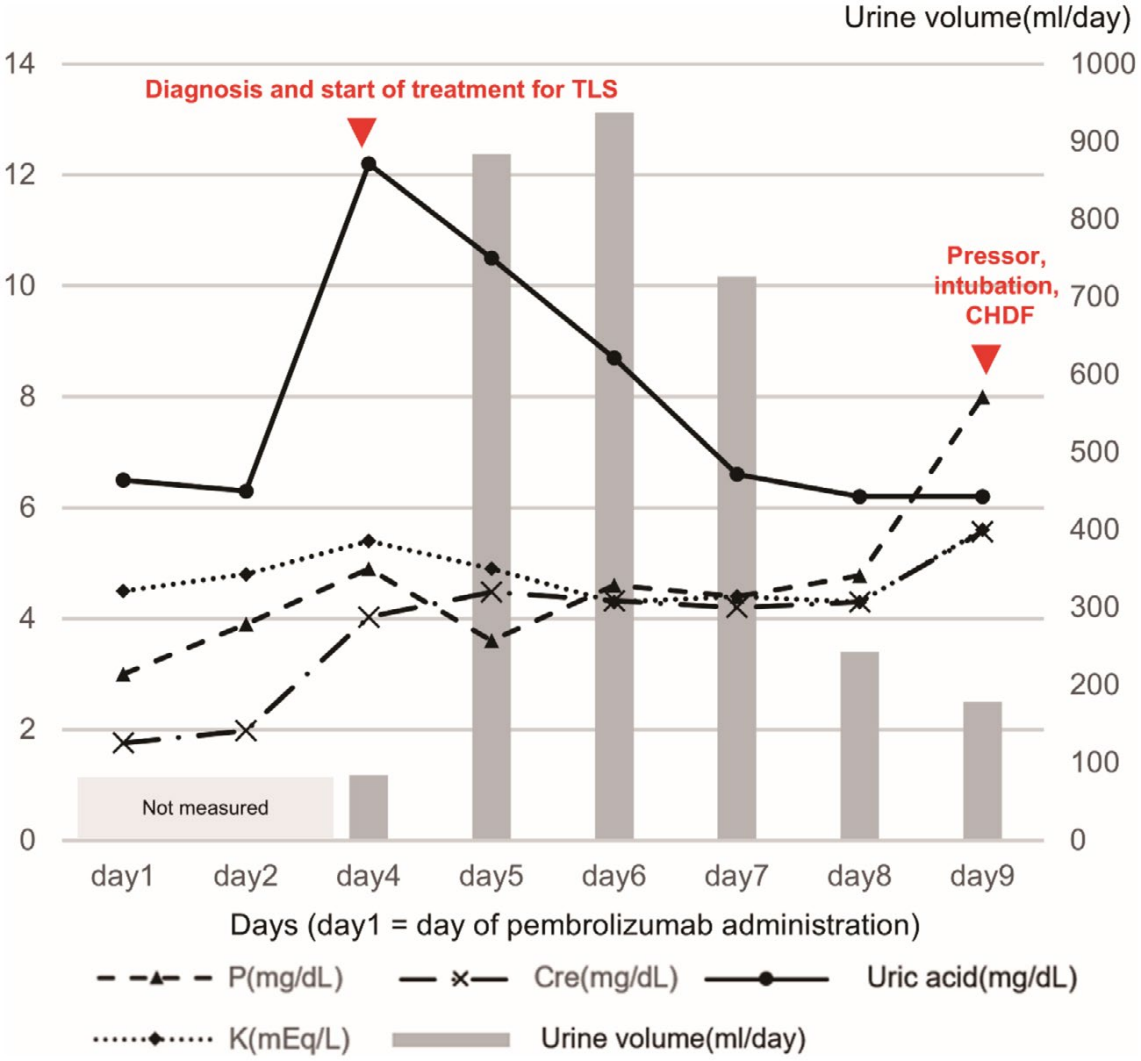
Clinical course. On day 4, the patient's uric acid and creatinine levels increased, with slight increases in potassium and phosphorus levels and the onset of oliguria. Treatment for TLS was initiated, which reduced uric acid levels and temporarily increased the urine output to 800 cc. However, the urine output decreased again on day 8. Creatinine levels showed no improvement after initiating TLS treatment.

Four days after the initiation of pembrolizumab, the patient developed fever and oliguria. His laboratory tests showed elevated uric acid and phosphorus levels, along with a sudden decline in renal function. Despite having high inflammatory markers (CRP 17.49 mg/dL), his CT revealed no infectious focus but did reveal progression of lung and liver metastases (Figure 1). Blood and urine cultures were negative for pyogenic bacteria. Moreover, ACTH, TSH, free T4, and cortisol levels (6.1 pg/mL, 2.61 μIU/mL, 1.02 ng/dL, 19.70 μg/dL, respectively) were normal, with no evidence of endocrine‐related irAEs. The patient was diagnosed with clinical TLS, which was characterized by hyperuricemia (12.2 mg/dL), hyperphosphatemia (4.03 mg/dL), and renal dysfunction (Cre, 4.03 mg/dL). The treatment management included febuxostat (60 mg) to inhibit uric acid production, 3 L of saline daily, and tazobactam/piperacillin (4.5 g) thrice daily. On the fifth day, the patient developed pulmonary edema and DIC, prompting the initiation of furosemide (20 mg). On the seventh day, his uric acid and electrolyte levels had normalized, and his urine output temporarily increased. However, the patient experienced a recurrence of oliguria, worsening pulmonary edema, and hypotension on the eighth day. The emergency measures on the ninth day included pressor agents, intubation, and continuous hemodialysis and filtration. Despite these intensive therapeutic interventions, the patient passed away on the following day. Figure 2 shows the changes in the urine volume and blood levels of creatinine, uric acid, potassium, and phosphorus throughout the hospitalization period. The temporal change in the DIC score is summarized in Table 1.

**TABLE 1:** DIC score during the clinical course.

Laboratory test (normal reference range)	Day‐5 (on admission)	Day 5	Day 7	Day 9
Plt (158–348 10^3^/μL)	280 [0]	119 [1]	95 [1]	117 [1]
FDP (0.0–4.9 μg/mL)	ND	ND^a^	102 [3]	80 [3]
Fib (193–412 mg/dL)	ND	120 [1]	140 [1]	80 [2]
PT ratio (0.85–1.15)	1.04 [0]	1.58 [1]	1.47 [1]	2.47 [2]
DIC score^b^	ND	ND	6	8

*Note:* Day 1 was defined as the day of pembrolizumab administration. The scores were calculated based on the 2017 DIC diagnostic criteria (basic type) for Japan [1]. Each subscore is shown in brackets [ ]. ND indicates “not determined”.

^a^ D‐Dimer was measured and the value was 139 ug/mL (0.00–0.99 ug/mL).

^b^ A score of 6 or more indicates a diagnosis of DIC.

## 3 Discussion

TLS represents a metabolic disturbance that arises from the extensive release of intracellular metabolites because of the rapid breakdown of tumor cells. This condition is characterized by hyperkalemia, which can potentially progress to fatal arrhythmias. Moreover, hyperuricemia and hyperphosphatemia can lead to renal tubule obstruction by uric acid and calcium phosphate crystals, thereby contributing to oliguric acute renal failure. In some instances, DIC may arise as a complication of cytokine storms, which are triggered by the release of intracellular cytokines [2, 3]. In our case, DIC was observed. Despite postpembrolizumab progression of lung/liver metastases, tumor‐associated DIC alone is unlikely to explain the hyperuricemia. However, the presence of acute hyperuricemia supports the manifestation of TLS‐related DIC, and the lack of an infectious focus with negative blood cultures makes infectious DIC unlikely. In 1993, Hande and Garrow initially proposed the diagnostic criteria for TLS [4]. In 2004, Cairo‐Bishop modified and divided these criteria into two stages: laboratory TLS and clinical TLS [5]. Laboratory TLS is diagnosed when two or more of the following electrolyte abnormalities are detected within 3 days before and 7 days after the initiation of treatment: hyperuricemia, hyperphosphatemia, hyperkalemia, and hypocalcemia. Meanwhile, clinical TLS is diagnosed if the patient also exhibits renal dysfunction, arrhythmia, convulsions, or sudden death [6].

TLS is frequently reported in hematological malignancies. However, it rarely occurs in solid tumors. A systematic review of studies from 1983 to 2020 identified 132 instances of TLS in solid tumors. Urological tumors accounted for 10 prostate cancer cases and six renal cell carcinoma cases. Among the cases of treatment‐induced TLS, 37% (49/132) manifested within 3 days of treatment administration, with a mortality rate of 54% (71/132) [7].

The reports of TLS in urological tumors treated with ICIs retrieved from PubMed, including our own cases, are summarized in Table 2. One case study reported a 67‐year‐old woman with metastatic urothelial carcinoma who developed TLS 8 days after atezolizumab administration [8]. Similarly, another report documented TLS that occurred 8 days after the initiation of combination therapy with pembrolizumab and axitinib in a patient with metastatic renal clear cell carcinoma [9]. Li Wang et al. comprehensively analyzed ICI‐related TLS using data from real‐world pharmacovigilance databases. Of the 164 cases that were retrieved, 23 cases (14%) were attributed to treatments targeting genitourinary conditions. The incidence of TLS associated with anti‐programmed death receptor‐1 (PD‐1) and anti‐programmed death ligand‐1 (PD‐L1) therapies has increased, from 10 cases in 2015 to 42 cases in 2020. The median time to onset for anti‐PD‐1/PD‐L1‐related TLS was 9 days (interquartile range, 2–40), with a mortality rate of 41% (47/113), underscoring the rapid onset and high fatality rate [10]. Chemotherapy‐induced TLS usually occurs within 48–72 h due to direct cytotoxicity [11], whereas ICI‐induced immune‐mediated tumor destruction may delay onset.

**TABLE 2:** Cases of TLS in urological tumors treated with immune checkpoint inhibitors.

Author	Cancer type	Matastatic lesion	Drug	Number of cycles to onset	Days to onset	Outcome
Faisal et al. [8]	Urothelial carcinoma	Lung, liver	Atezolizumab	1	8	Shifting to palliative care
Manan et al. [9]	Renal cell carcinoma	Lung, liver	Pembrolizumab and axitinib	1	8	Death
Our case	Urothelial carcinoma	Left adrenal gland, liver, lung, para‐aortic lymph nodes	Pembrolizumab	1	4	Death

Gemici et al. identified several risk factors for TLS in solid tumors, including large tumor volume (≥ 10 cm), liver metastases, elevated LDH or uric acid levels, high susceptibility to chemotherapy (e.g., neuroblastoma, germ cell tumors, and small‐cell lung cancer), renal dysfunction, use of nephrotoxic drugs, and co‐existing infection or dehydration [12]. In Japan, the TLS treatment guidelines generally classify solid tumors as low risk for TLS development; however, they are reclassified as intermediate risk when one or more of the aforementioned risk factors are present [2]. Stringent management through frequent blood sampling and monitoring, along with the oral administration of febuxostat starting one to 2 days before treatment, is advised for intermediate‐risk cases. In addition, high‐volume saline infusion is recommended as a preventive measure [2]. The severity of TLS might have been reduced if hydration or a uric acid oxidase inhibitor had been utilized before treatment rather than after onset. Upper urinary tract tumors often cause renal dysfunction, and routine prophylaxis in all cases may constitute overtreatment. Here, renal dysfunction with LDH > 1000 U/L and liver metastases justified prophylaxis when multiple additional risk factors existed.

## 4 Conclusion

This case report presents a fatal episode of TLS following pembrolizumab administration in which prophylactic measures prior to treatment were likely inadequate. Given the poor prognosis of TLS, such interventions should be considered for patients at intermediate risk, especially when multiple non‐renal risk factors exist.

## Ethics Statement

The authors have nothing to report.

## Consent

The authors have nothing to report.

## Conflicts of Interest

The authors declare no conflicts of interest.
